# Comparing the Perceptions of Reciprocal- and Near-Peer Objective Structured Clinical Examinations (OSCEs) in Medical Students

**DOI:** 10.7759/cureus.35535

**Published:** 2023-02-27

**Authors:** Olivia Calisi, Steven King, Daniel J Berger, Munima Nasir, Sarah Nickolich

**Affiliations:** 1 Medicine, Penn State University College of Medicine, Hershey, USA; 2 Department of Family and Community Medicine, Penn State University College of Medicine, Milton S. Hershey Medical Center, Hershey, USA

**Keywords:** near peer teaching, reciprocal peer teaching (rpt), medical school education, preclinical medical students, first year medical students, objective structured clinical exam (osce)

## Abstract

Introduction: The Objective Structured Clinical Examination (OSCE) is utilized by medical schools to assess students’ competency in clinical skills. Literature has shown that first-year students who were tutored by fourth-year students (MS4s; near-peer) in practice OSCEs reported self-perceived improvement in OSCE skills. There is a lack of research regarding the effectiveness of first-year (MS1) pairs for reciprocal-peer practice OSCEs. This study aims to assess if virtual reciprocal-peer OSCEs provide comparable learning opportunities to virtual near-peer OSCEs.

Methods: MS1 students were assigned to work with a near-peer or a reciprocal-peer for one week, and then switched protocols the second week. One student in each reciprocal-peer pair was assigned to act as a standardized patient (SP). Their partner took a history, interpreted physical exam findings, prepared a note, and gave an oral presentation. The pair then switched roles using a second case. The near-peer group followed the same procedure, without the reversal of roles.

Results: A total of 135 MS1s participated in the first week and 129 in the second week. Students agreed that working with a near-peer was more valuable than a reciprocal-peer in the following parameters: peer feedback (N=113, 89%), history-taking skills (N=101, 80%), physical exam skills (N=102, 81%), and note-writing skills (N=109, 89%). Pairwise comparison utilizing Wilcoxon signed-rank test indicated participants preferred the choice of a fourth-year student partner over an MS1 partner (Z=1.436, p<0.001).

Conclusion: Participants found working with a near-peer increased confidence in their clinical skills and near-peer feedback was more valuable. Although MS1s found that watching and evaluating their peers in a reciprocal-peer exercise was beneficial, students overwhelmingly preferred working with MS4s due to more valuable feedback.

## Introduction

The Objective Structured Clinical Examination (OSCE) is used to assess clinical skill performance and competence by various healthcare professional schools [1]. There are various modalities of OSCE protocol. For example, in the United Kingdom, the OSCE is designed as a single-station exercise where students are expected to perform one component of the clinical encounter [2]. In contrast, in the United States, the OSCE mimics the protocol of Step 2 Clinical Skills (Step 2 CS), a United States Medical Licencing Examination [2]. In this situation, students simulate a clinical encounter and are asked to perform a variety of clinical skills (interviewing a standardized patient (SP), performing a clinical exam, creating a list of differential diagnoses, and writing a note) in a set period of time [2,3]. They are subsequently graded by evaluators on their performance [3]. With the cancellation of Step 2 CS, the responsibility for assessing clinical skill readiness falls further onto medical schools. With this change, OSCEs become a more essential curricular component. However, students report these examinations to be stressful [4,5]. Although stress and anxiety levels were not predictive of performance outcome, perceived confidence in one's capabilities has been shown to correlate with performance [4,5].

One way to increase perceived confidence in OSCE skills is through peer tutoring. Research has established that junior medical students benefit from working with senior medical students (near-peer) during practice OSCEs. Specifically, junior medical students reported feelings of self-perceived improvement in preparation for and performance in summative OSCEs after working with a near-peer. In some studies, near-peer tutors were found to be as effective as faculty tutoring [6,7], while one study found that near-peers were preferred and more effective than faculty teaching [8].

Prior studies investigated the use of near-peer tutoring in medical schools both in anatomy curricula and practice OSCE scenarios [9-11]. Near-peer tutoring is a mutually beneficial learning strategy, as the tutor experiences learning benefits such as review of concepts, practice of clinical skills, and practice providing feedback [12-14]. Near-peer learning has also been shown to be an effective alternative in times of stretched faculty resources [12,13].

As with near-peer tutoring, research has established that tutoring with reciprocal-peers, or students in the same cohort, benefits both the tutor and tutee [9]. However, there is a lack of research regarding the efficacy of pairing first-year medical students with other first-year medical students (MS1-MS1) for reciprocal-peer practice OSCEs. This study hopes to determine if reciprocal-peer virtual practice OSCEs are perceived as valuable experiences for reviewing clinical skills such as history-taking, physical exam interpretation, note writing, and oral presentations. Additionally, it will assess if first-year medical students consider working with their reciprocal peer partner as valuable as working with their near-peer partner.

This article was previously presented as a meeting abstract at the following meetings: Pennsylvania Academy of Family Physicians on March 13, 2021, EdVenture at Penn State College of Medicine on April 29, 2021, and Innovations in Medical Education Conference on February 17, 2022.

## Materials and methods

The student sample

MS1s were randomly divided into two student groups, with one group performing the reciprocal-peer OSCE protocol (MS1-MS1) and the other performing the near-peer OSCE protocol (MS1-MS4) in the first week. The students switched protocols the following week. MS4 students were recruited from the Students as Educators (SaE) Pathway, a longitudinal clinical teaching elective at Penn State College of Medicine. Due to COVID-19 restrictions, OSCEs were conducted over Zoom (Zoom Video Communications, Inc, San Jose, CA, USA), a video conferencing medium.

The formative OSCE protocol

Virtual practice OSCEs were conducted over a two-week period. Six clinical cases were divided between the reciprocal-peer and the near-peer OSCE protocols.

In the reciprocal-peer protocol, one student was assigned to act as the SP and given a packet with instructions and the clinical case. Their partner was designated 20 minutes to conduct a history and interpret physical exam findings. Since the sessions were conducted over Zoom, the SPs verbalized the physical exam results (ex. "heart rate is slightly tachycardia but with a regular rhythm, no murmurs, no rubs, no gallops."). In addition, the SP displayed photos that corresponded to the case’s physical examination findings. The student was given 20 minutes to prepare a note, followed by a five-minute oral presentation. The SP then provided 15 minutes of formative feedback. The pair then switched roles using a second case. The near-peer group followed the same procedure for one case where the MS4s acted as the SP and provided feedback.

To ensure a comparable educational experience, the groups were switched the following week so all MS1s would have the opportunity to work with both an MS1 and MS4. Alternative clinical cases were utilized during the second week to ensure they would be new to all participants.

Evaluation strategy

Sessions were evaluated after completion to determine perceptions of confidence and value via surveys consisting of seven-point Likert scale questions and short-answer responses. In total, each participant was administered two surveys for a total of 24 questions. Study data were collected and managed using Research Electronic Data Capture (REDCap) electronic data capture tools hosted at Penn State Health Milton S. Hershey Medical Center and Penn State College of Medicine [15,16]. REDCap is a secure, web-based software platform designed to support data capture for research studies, providing 1) an intuitive interface for validated data capture; 2) audit trails for tracking data manipulation and export procedures; 3) automated export procedures for seamless data downloads to common statistical packages; and 4) procedures for data integration and interoperability with external sources.

Separate, non-anonymous data were recorded to ensure the completion of these sessions for academic credit. The study was submitted to the Pennsylvania State University College of Medicine Institutional Review Board for review and was given an exemption determination.

Analysis

Data analysis consisted of descriptive and nonparametric tests in SPSS Statistics version 27 (IBM, Armonk, NY, USA). A Mann-Whitney U Test was used to assess student perceptions of their confidence in clinical skills and the value of feedback after their experience with a near- or reciprocal-peer. A Friedman Test was used to analyze student preference for either an SP, near-peer, or reciprocal-peer. Post hoc analysis with Wilcoxon Signed Ranks Test with a Bonferroni correction was conducted, resulting in a significance level set at p<0.017.

## Results

During the first week, 135 students participated in the virtual OSCE. Of those 135 students, 66 participated in the reciprocal-peer experience and 69 participated in the near-peer experience. In the second week, 129 students participated in the OSCE. Of these, 64 were in the reciprocal-peer group while 65 were in the near-peer group. 

Reciprocal-peer experience

Students who participated in the reciprocal-peer experience largely agreed that it improved their confidence and the feedback was valuable. Table 1 demonstrates the percent of students in each week who indicated that they agreed to some degree (somewhat agree, agree, strongly agree) that the reciprocal-peer OSCE improved their confidence in their clinical skills and that receiving feedback was valuable.

**Table 1:** Student agreement to survey statements following participation in the reciprocal-peer experience. All statements are in reference to their reciprocal-peer experience. OSCE: Objective Structured Clinical Examination

	Week 1 (N=66)		Week 2 (N=64)	
Statement	N	%	N	%
Participation helped me understand OSCE assessment goals:	54	82.0%	55	86.0%
Participation enhanced my clinical reasoning ability:	51	77.0%	53	83.0%
Participation improved my confidence in my ability to take history:	51	77.0%	57	89.0%
Participation improved my confidence in physical exam skills:	41	62.0%	45	70.3%
Participation improved my confidence in my ability to write a note:	49	74.0%	55	86.0%
Participation improved my confidence to give an oral presentation:	47	71.0%	51	80.0%
Getting feedback on my patient interview was a good experience:	49	74.0%	48	75.0%
Getting feedback on my note was a good experience:	40	61.0%	42	66.0%
Getting feedback on my oral presentation was a good experience:	39	59.0%	45	70.3%
Getting feedback on my clinical reasoning was a good experience:	41	62.0%	42	66.0%
Participation in the Reciprocal-Peer OSCE was a good experience:	47	71.0%	53	83.0%

A majority of students in each week agreed that the reciprocal-peer OSCE improved their confidence in their clinical skills and that the feedback from their reciprocal peer was valuable. Student perceptions of their role as the SP/tutor were also provided. Students largely agree that acting as the SP helped them understand OSCE assessment goals and clinical skills (Table 2). When asked if participating in a reciprocal-peer OSCE was valuable overall to their learning, students in week one (71%, N=47) and week two (79%, N=51) agreed that it was to some degree.

**Table 2:** Student perceptions of how observing their peer and acting in the SP/tutor role helped them understand OSCE goals and clinical skills. SP: Standardized Patient; OSCE: Objective Structured Clinical Examination

	Week 1 (N=66)		Week 2 (N=64)	
Statement	N	%	N	%
Participation as an SP helped me understand OSCE assessment goals:	54	82.0%	53	83.0%
Participation as an SP enhanced my ability to provide feedback to peers:	52	79.0%	53	83.0%
Participation as an SP helped me improve my ability to take a history:	54	82.0%	57	89.0%
Participation as an SP helped me improve my physical exam skills:	49	75.0%	45	70.3%
Participation as an SP helped me improve my ability to write a note:	45	68.0%	55	86.0%
Participation as an SP helped me improve my oral presentation:	48	73.0%	51	80.0%

Near-peer experience

Students who participated in the near-peer experience largely agreed that it improved their confidence and that the feedback from their peer was valuable. Table 3 demonstrates the percentage of students in each week who indicated they agreed to some degree (somewhat agree, agree, strongly agree) that the near-peer OSCE improved their confidence in their clinical skills.

**Table 3:** Student perceptions of confidence in clinical domains and value of feedback following participation in near-peer OSCEs. OSCE: Objective Structured Clinical Examination

	Week 1 (N=69)		Week 2 (N=65)	
Statement	N	%	N	%
Participation helped me understand OSCE assessment goals:	63	91.0%	61	94.0%
Participation enhanced my clinical reasoning ability:	64	93.0%	60	92.0%
Participation improved my confidence in my ability to take a history:	66	96.0%	60	92.0%
Participation improved my confidence in my physical exam skills:	52	75.0%	55	85.0%
Participation improved my confidence in my ability to write a note:	63	91.0%	60	92.0%
Participation improved my confidence to give an oral presentation:	65	94.0%	60	92.0%
Getting feedback on my patient interview was a good experience:	67	97.0%	62	95.0%
Getting feedback on my note writing skills was a good experience:	64	93.0%	59	91.0%
Getting feedback on my oral presentation was a good experience:	67	97.0%	61	94.3%
Getting feedback on my clinical reasoning was a good experience:	67	97.0%	60	92.0%
Participation in the Reciprocal-Peer OSCE was a good experience:	62	90.0%	61	94.0%

A majority (>90%) of students agreed that the near-peer experience improved their confidence in their clinical skills and that the feedback provided was valuable. The only domain where less than 90% of students agreed was in their confidence in performing physical exams. Importantly, when asked if participating in near-peer OSCEs was valuable for their overall learning, students in week one (90%, N=62) and week two (94%, N=61) agreed it was.

Comparison between near- and reciprocal-peer experiences

**Figure 1:**
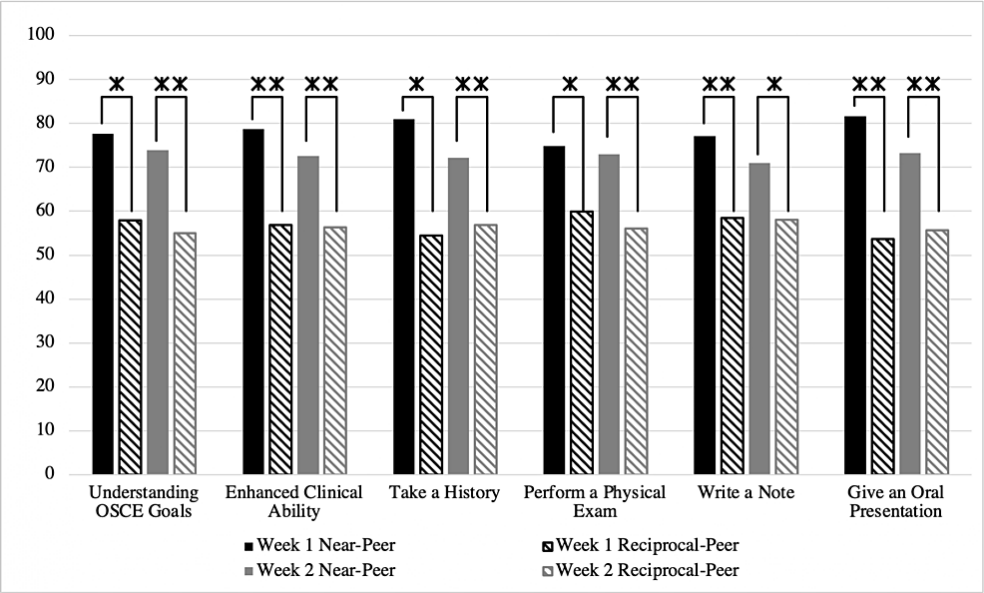
Perceived confidence in learning domains following near and reciprocal-peer experiences, evaluated using Mann-Whitney U test. The y-axis indicates mean ranks. *Indicates statistical significance at p<.05 statistical significance at p OSCE: Objective Structured Clinical Examination

Student perceptions of confidence in clinical skills and the value of feedback received following their experiences with near- and reciprocal-peers were compared. Figure 1 demonstrates the results of a Mann-Whitney U test performed to evaluate student confidence levels in clinical domains.

**Figure 2:**
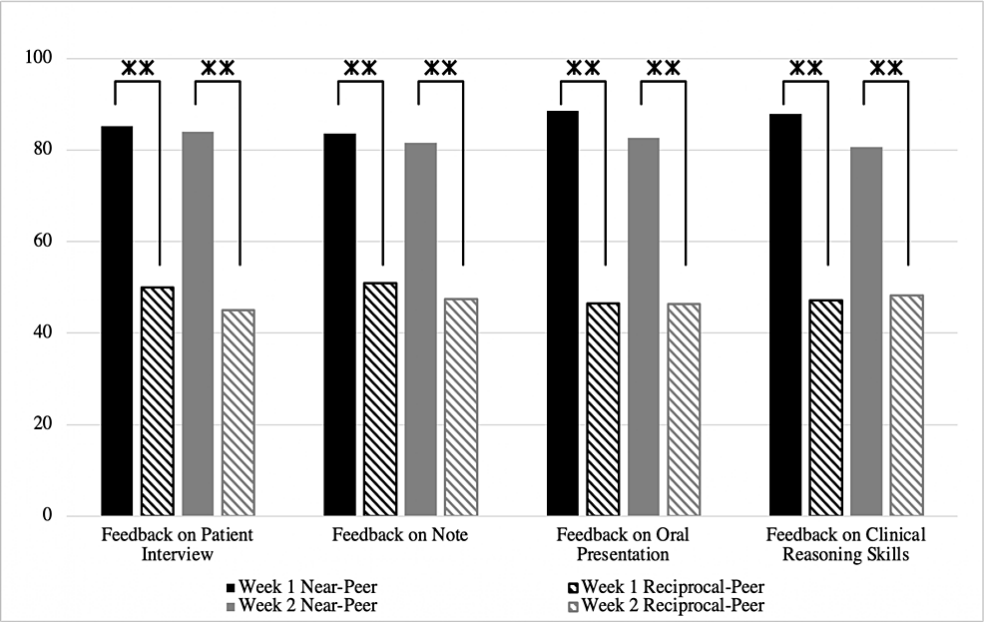
Perceptions of value regarding feedback from an MS4 or MS1 partner in weeks one and two. The y-axis indicates mean ranks. **Indicates statistical significance at p<.01 MS1: first-year; MS4: fourth-year

In all cases related to confidence in clinical skills, there were statistically significant differences between near- and reciprocal-peer experiences, in favor of near-peer over reciprocal-peer. In addition, a comparison was done between the perceived value of feedback. Figure 2 demonstrates statistically significant differences in all domains, indicating that students found feedback from their near-peer to be more valuable.

In addition, a total of 126 students answered questions comparing their experiences with their reciprocal-peer and near-peer. Students agreed that working with a near-peer was more valuable than a reciprocal-peer in the following parameters: peer feedback (N=113, 89%), history-taking skills (N=101, 80%), physical exam skills (N=102, 81%), and note-writing skills (N=109, 89%).

Students ranked their preference for working with a reciprocal-peer (MS1-MS1), near-peer (MS4-MS1), and a theoretical SP. Analysis with a Friedman Test indicated a statistically significant difference between the preferred type of partner, χ2(2) = 179.949, p<0.001, W=.818. Pairwise comparison utilizing Wilcoxon signed-rank test indicated participants preferred the choice of MS4s over MS1s (Z=1.436, p<0.001) and SPs over MS1s (Z=1.182, p<0.001). There was no preference between MS4s and SPs (Z=-2.255, p=0.177).

Student qualitative perceptions

A total of 101 students answered optional qualitative free-response questions. Several themes arose from the answers that highlight the students’ perceptions regarding the exercise.

Most students reported receiving feedback as the most helpful part of the activity for their learning. Notably, 34% of the students specified that the feedback from the MS4 was helpful due to the senior student’s clinical experience and credibility.

“[the most valuable part of this exercise was] receiving feedback from a 4th year who has already gone through their 3rd-year clerkships and can tell us what to accept/what we need to improve to be ready for 3rd year in terms of our history taking and oral presentations." 

"My MS4 was particularly helpful in how to write the note properly and how to improve my oral presentation. She made me feel good about my performance as well as giving helpful feedback."

"The MS4 was reassuring that my clinical reasoning with regard to the DDX [differential diagnosis] was at the point it should be during our medical education."

Some students stated that the reciprocal-peer aspect was beneficial as it helped them reflect on their own abilities as well as understand the requirements of the OSCE.

“The most helpful part of this remote OSCE was being able to compare myself to my reciprocal peer. During this exercise, we were able to watch and learn from each other to see what we both did well and what we both could improve upon.”

“Being able to act as an SP and see the kind of information we need to get from the student physician side was really helpful for me”

“Seeing the rubric was good for gauging what was expected of us”

"It was helpful to watch someone else take a history as it helped me understand how I could improve upon my own history taking. I also liked seeing how other students build rapport with their patients."

“It was helpful to be the standardized patient, as I can tailor my responses to what the physician asks, which helps me understand as the student physician, how to ask the questions to make sure I can cover everything.”

However, some students were more critical regarding the reciprocal-peer OSCE.

"I preferred the near-peer remote OSCE because the peer-to-peer one felt more like the blind leading the blind when it came to giving feedback."

"It's hard to provide feedback as an MS1. I would recommend having OSCEs with someone more knowledgeable like a professor or MS4. I don't know how much I was able to help my partner because my knowledge feels very limited when it comes to narrowing the physical exam and diagnoses.”

In addition, students stated that the practice of the OSCE skills was a key benefit of this experience, noting that both the practice and feedback helped to boost confidence in their skills.

“Every time I get to practice taking a history and thinking about a differential is helpful. Just going through those motions builds my confidence and helps me reflect on areas to improve. It is helpful to practice with a reciprocal peer to have a more relaxed environment, but the best feedback comes from an SP/SAC [Society Advisor Coach or physician faculty member]. The MS4 session was a nice balance of both. I enjoyed experiencing all three of these settings to practice.”

## Discussion

With the cancellation of USMLE Step 2 CS, and therefore a greater responsibility for medical schools to assess their student’s clinical competency, OSCEs can provide both practice and a way to evaluate students. There are various approaches to OSCEs in the curriculum, including the use of SPs, near-peers, and reciprocal-peers. To our knowledge, this is the first study that directly compares near-peer to reciprocal-peer learning for OSCE preparation in the American medical school setting.

When evaluating the OSCE experiences separately, there are benefits to each. Students largely agreed that reciprocal-peer OSCEs were valuable to their learning and enhanced their confidence in their clinical skills. In particular, they agreed that the experience of acting as an SP and a grader allowed them to understand the grading criteria, provide feedback, and improve OSCE-related skills. Notably, observing their MS1 partners fostered self-reflection regarding their own abilities and allowed them to gain insight into what type of information a student physician should seek in a clinical encounter. Furthermore, students enjoyed the reciprocal-peer environment and noted they were comfortable working with their reciprocal-peer partner. Overall, the primary advantage of the reciprocal-peer modality is allowing students to participate in both roles of the OSCE.

When it came to working with near-peers, students were overwhelmingly pleased with their experience. In all clinical domains such as history taking, physical exam skills, note writing, and giving oral presentations, students felt their confidence improved and that the feedback was valuable. This is consistent with previous research, which has also demonstrated the success of near-peer experiences in improving confidence [6,17]. Students’ short-answer responses indicated that the feedback from near-peers was helpful. MS4 students were perceived as being more knowledgeable due to their prior clinical clerkship experiences. In addition, the near-peers made students feel comfortable by providing reassurance that their clinical skills were adequate for their level of training. Ultimately, students found more value in feedback from their fourth-year counterparts compared to their first-year peers, which likely influenced their self-perceived confidence in clinical skills.

The direct comparison between near- and reciprocal-peer OSCEs indicated that students found more benefit in working with near-peers. While our study demonstrates students generally agree both experiences improved their confidence and that the feedback was valuable, the near-peer experience outperformed the reciprocal-peer experience in both of these areas. Students found more value in working with a near-peer on their history-taking, physical exam, and note-writing skills. Perceived confidence in these skills was also greater in the near-peer group. In addition, students stated it was more valuable to receive feedback from a near-peer in each of these domains. Furthermore, when directly asked about which experience students preferred, they specified they would rather work with a near-peer over a reciprocal-peer. It is important to note, that even with the apparent benefit of having a student play both roles in the reciprocal-peer experience, students still found that the near-peer experience was more valuable. Given these results, near-peer is viewed by students as the superior learning modality.

The apparent preference for near-peers can likely be explained by examining how students perceived feedback. Students were more skeptical of the feedback they received from a reciprocal-peer, suggesting they doubted the legitimacy of the advice. Particularly, students worried that without clinical experience from clerkships, the feedback from a reciprocal-peer would not be useful. In addition, students reflected that even as an SP and grader, they struggled to provide feedback to a peer. This is consistent with prior literature, where the disadvantage of reciprocal-peer tutoring is the quality of teaching from their peers [9]. Moreover, in a study evaluating residents, Bing-You (1997) found that residents were more likely to discount feedback from an evaluator who they thought had a low level of knowledge or lacked experience as a physician [18]. This is an inherent disadvantage of utilizing reciprocal-peers compared to near-peers, and can help explain our findings.

Taking these findings into account, both reciprocal-peer and near-peer experience may be beneficial to the medical school curriculum. The advantages of the reciprocal-peer experience include an introduction to the OSCE process, practice with OSCE-style cases, and a comfortable learning environment with their peers. Therefore, having students participate in a reciprocal-peer OSCE early in their medical school experience could effectively clarify how OSCEs are conducted and how students should expect to be assessed. This greater understanding of the OSCE process may be useful to students in their future OSCEs, although this is something that could be examined in a future study. In terms of a near-peer experience, this can occur later in the medical school curriculum. Given that our findings suggested that students had no preference between an SP and a fourth-year medical student, the fourth-year medical student may replace the SP in a summative OSCE experience. In addition, students found the feedback from their near-peer to be very valuable. Therefore, it could be worthwhile to utilize a near-peer experience after students have developed a deeper understanding of how OSCEs are conducted.

While the data provide insight into the comparison of near-peer and reciprocal-peer learning opportunities, there are certain limitations in the study. First, the MS4s who participated in this study were volunteers from the optional “Students as Educators” elective and received specific training regarding teaching methodology, evaluation, and providing feedback. Students who volunteer for teaching are identified to have an interest in medical education and are not representative of all fourth-year medical students. In addition, first-year students were not provided the equivalent instruction as the MS4s. Secondly, we did not collect data according to which cases were assigned. Participant survey results may have been correlated to specific cases that were utilized. Thirdly, MS1s had one formative virtual OSCE with an SP prior to this experience. Although they had a basic understanding of the role of the student doctor and the SP, their limited experience with OSCEs could significantly affect their abilities to act as an SP or provide constructive feedback. The virtual format also made it difficult for students to practice physical exam skills. Our results regarding perceived confidence in physical exam skills may not be comparable to an experience with in-person settings.

## Conclusions

This study evaluated the role of near-peer and reciprocal-peer virtual OSCEs as an educational tool in the medical school curriculum. While both experiences led to increased confidence in clinical skills and valuable feedback, students indicated that working with a near-peer increased their perceived confidence in various domains, such as note-writing ability and clinical reasoning more than working with a reciprocal-peer. Additionally, students felt that the feedback given to them by their near-peer was more valuable than that from the reciprocal-peer. A qualitative assessment revealed that participants perceived near-peers as more knowledgeable compared to their reciprocal-peer, which could account for their preference. However, first-year students agreed that serving in the tutor role and providing feedback to their peers aided in their learning. Future studies involving an in-person reciprocal- vs. near-peer OSCE may be beneficial, as students could more effectively practice their physical exam skills. Additionally, future studies may want to expand upon qualitative aspects of this study to further understand the reasoning for student preference. It is also worthwhile to investigate the actual OSCE performance of students after they have served in a tutor role and have provided feedback to their peers.

## Figures and Tables

**Figure 3:**
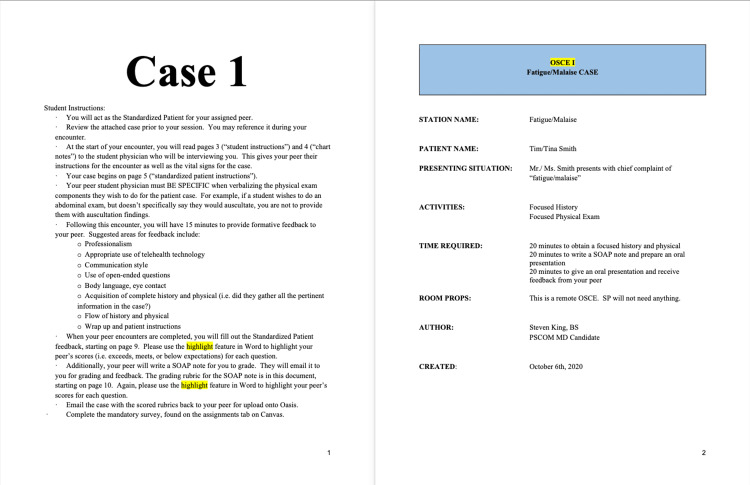
Pages 1 and 2 of Standardized Patient OSCE (Objective Structured Clinical Examination) Guide

**Figure 4:**
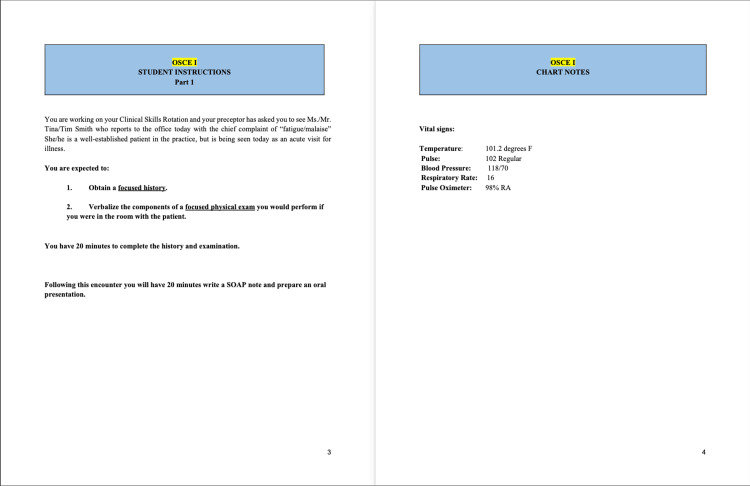
Pages 3 and 4 of Standardized Patient OSCE (Objective Structured Clinical Examination) Guide

**Figure 5:**
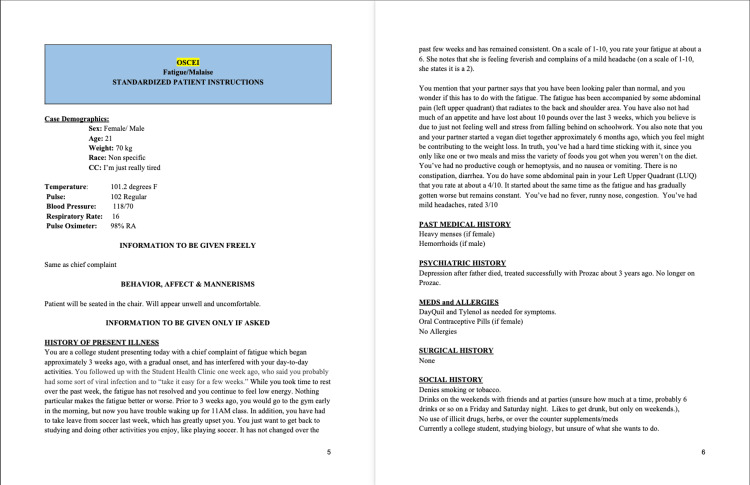
Pages 5 and 6 of Standardized Patient OSCE (Objective Structured Clinical Examination) Guide

**Figure 6:**
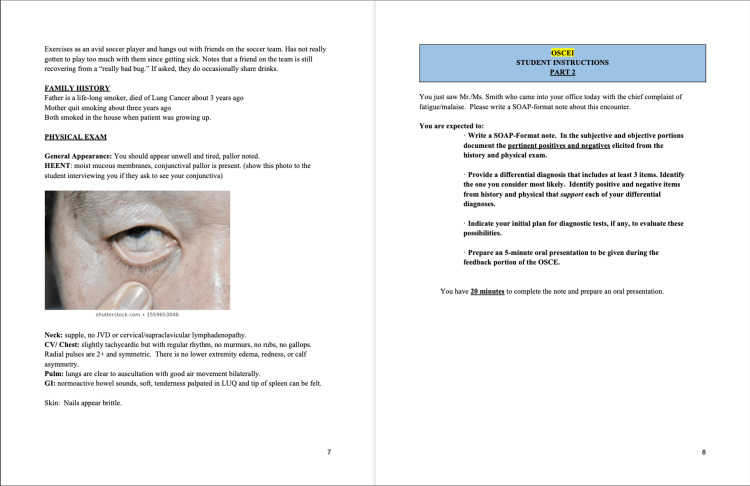
Pages 7 and 8 of Standardized Patient OSCE (Objective Structured Clinical Examination) Guide

**Figure 7:**
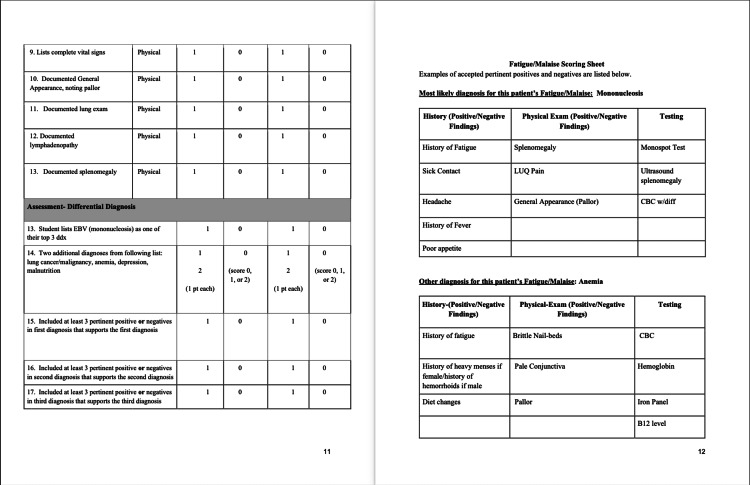
Pages 11 and 12 of Standardized Patient OSCE (Objective Structured Clinical Examination) Guide

**Figure 8:**
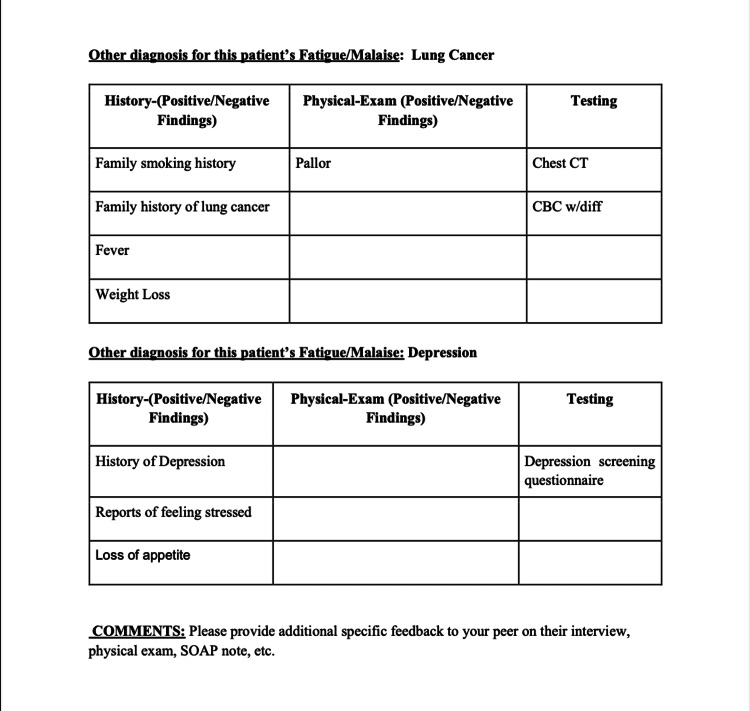
Page 13 of Standardized Patient OSCE (Objective Structured Clinical Examination) Guide
